# Ankylose de l'articulation temporo-mandibulaire post-arthrosique

**DOI:** 10.11604/pamj.2019.32.151.17779

**Published:** 2019-03-28

**Authors:** Abdelouahid Taleuan, Dounia Kamal, Lotfi Aouinti, Moahamed Nourdine Elalami

**Affiliations:** 1 Service d'Oto-Rhino-Laryngologie et Chirurgie Cervico-Faciale, CHU Hassan II, Fès, Maroc Maroc

**Keywords:** Articulation temporo-mandibulaire, arthrose, ankylose, Temporomandibular joint, arthrosis, ankyloses

## Abstract

L'ankylose de l'articulation temporo-mandibulaire (ATM) est définie comme une constriction permanente des mâchoires avec ouverture buccale inférieure à 30mm mesurée entre les incisives, survenant en raison d'une fusion osseuse, fibreuse ou fibro-osseuse. L'arthrose est une cause rare de l'ankylose de L'ATM. Nous rapportons un cas d'ankylose de l'ATM d'origine arthrosique, afin de préciser les particularités diagnostiques et thérapeutiques de cette entité pathologique assez rare.

## Abstract (en)

Ankylosis of the temporomandibular joint (TMJ) is a joint stiffness with an oral aperture of less than 30 mm measured between the incisors, occurring because of a bony, fibrous or fibro-osseous fusion. Arthrosis is a rare cause of the ankylosis of the temporomandibular joint. We report a case of ankylosis of the TMJ due to osteoarthrosis, in order to highlight the diagnostic and therapeutic features of this quite uncommon disease.

## Introduction

L'ankylose de l'articulation temporo-mandibulaire (ATM) est définie comme une constriction permanente des mâchoires avec ouverture buccale inférieure à 30mm mesurée entre les incisives, survenant en raison d'une fusion osseuse, fibreuse ou fibro-osseuse [1]. L'arthrose de l'ATM est une maladie dégénérative, affectant progressivement le cartilage, la membrane synoviale et les structures osseuses. À un stade avancé, il en résulte de graves dommages aux structures de l'ATM voir un développement d'ankylose [2]. Nous rapportons un cas d'ankylose de l'ATM d'origine arthrosique afin de préciser les particularités diagnostiques et thérapeutiques de cette entité pathologique assez rare.

## Patient et observation

**Figure 1:**
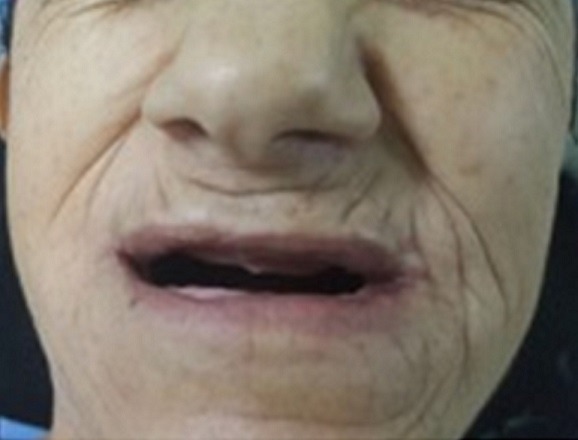
Photo préopératoire de la patiente montrant un trismus serré

**Figure 2:**
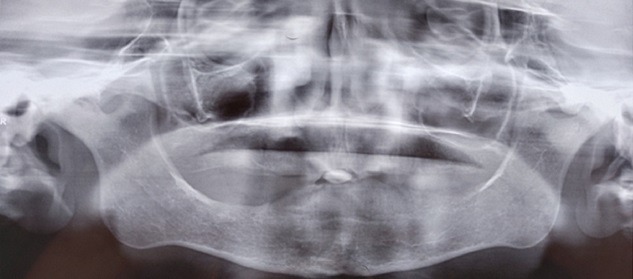
Radiopanoramique dentaire montrant un pincement irrégulier de l’interligne articulaire avec aplatissement condylien bilatéral

**Figure 3:**
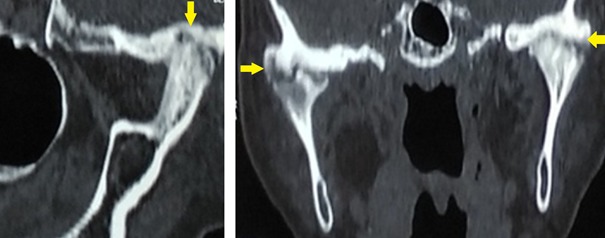
TDM (tomodensitométrie) en coupes coronale, sagittale montrant une ankylose de l’ATM bilatérale avec des géodes et des ostéophytes

**Figure 4:**
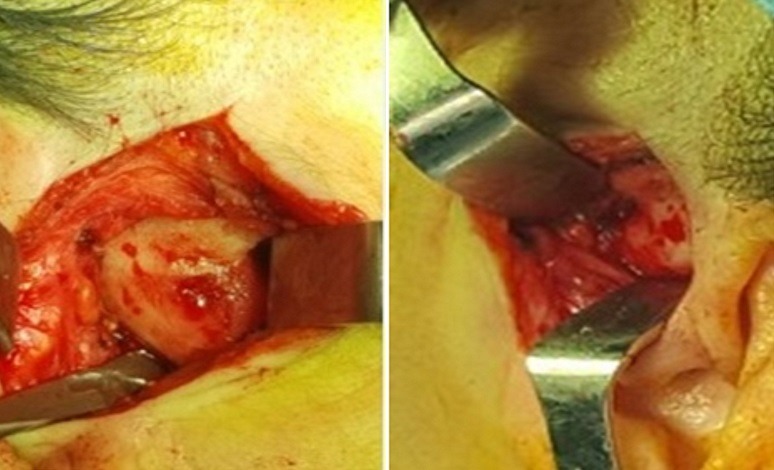
Images per-opératoires montrant le bloc de l´ankylose de l´ATM en bilatéral après un abord de Ginestet

**Figure 5:**
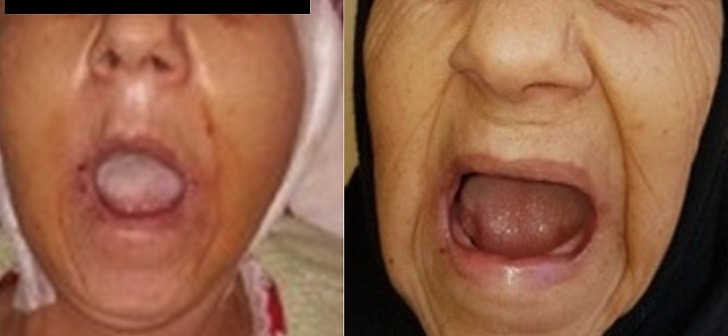
Images postopératoires immédiate et tardive montrant une nette amélioration du trismus avec un gain de 3cm

Il s'agit d'une patiente âgée de 57 ans, sans notion de traumatisme cranio-facial, qui présente depuis trois ans avant son admission des craquements au niveau des deux ATM avec des douleurs bilatérales pré-tragiennes plus exagérées le soir; la symptomatologie s'est aggravée deux ans après par l'apparition d'une limitation de l'ouverture buccale devenant de plus en plus invalidante, le tout évoluant dans un contexte d'apyrexie et de conservation de l'état général. A l'examen, la patiente était apyrétique, édentée et présentait un trismus serré gênant l'examen endobuccal (Figure 1). Les aires ganglionnaires cervicales étaient libres. L'examen otoscopique et rhinoscopique était sans particularité. L'orthopantomogramme (Figure 2) a montré un pincement bilatéral de l'interligne des surfaces articulaires qui étaient irrégulières avec aplatissement condyliens, des ostéophytes temporo-mandibulaires et une déminéralisation osseuse. Le bilan inflammatoire à base de vitesse de sedimentation (VS) et protéine C-réactive (CRP) était négatif. Un complément scannographique de la face a mis en évidence des géodes sous chondrales au niveau des condyles mandibulaires, avec aplatissementdes fosses temporo-mandibulaires et néo-ossification de la capsule et des ménisques articulaires réalisant une importante ankylose des ATM (Figure 3). Ces données anamnestiques, cliniques et radiologiques nous ont permis d'imputer l'ankylose à l'arthrose de L'ATM. La patiente a bénéficié d'une résection bilatérale du bloc ankylosique après un abord du Ginestet (Figure 4) suivie d'un coronoidectomie bilatérale par voie vestibulaire avec obtention en peropératoire d'une bonne ouverture buccale. Les suites opératoires étaient simples. La patiente a bénéficié d'une rééducation fonctionnelle précoce et prolongée pendant six mois. Les résultats ont été satisfaisants (Figure 5) avec un gain de 3cm d'ouverture buccale après un recul de 1 an et demi.

## Discussion

L'ankylose de l'ATM est une pathologie grave et invalidante par ses répercussions fonctionnelles sur la mastication, l'hygiène bucco-dentaire, la respiration, la phonation, mais aussi morphologiques et psychologiques. Son diagnostic est clinique et sa confirmation est radiologique [3]. Sur le plan étiopathogénique, l'ankylose est toujours secondaire, avec par ordre de fréquence: les traumatismes (y compris iatrogènes), les infections et les inflammations. Les causes inflammatoires regroupent essentiellement la polyarthrite rhumatoide, la spondylarthrite ankylosante, la polyarthrite chronique juvénile (maladie de Still) et le rhumatisme psoriasique [1]. La société française de rhumatologie définit l'arthrose comme une pathologie qui conduit à une destruction plus ou moins importante des surfaces articulaires pouvant entrainer des poussées inflammatoires secondaires. C'est pourquoi le terme d'arthrite chronique dégénérative est un synonyme d'arthrose. Des études ont révélé que l'arthrose est plus fréquente chez la femme et que l'âge moyen d'apparition de l'arthrose de l'ATM est de 35 ans. Ce qui correspond à une apparition 10 années plus tôt, par rapport à d'autres articulations, telles que celle du genou [4]. Dans la majorité des cas, l'arthrose est plurifactorielle. Elle regroupe des facteurs locaux (traumatismes, parafonctions, surcharges articulaires) et généraux (âge, sexe, hérédité). Les contraintes mécaniques exercées sur l'articulation tout au long de la vie (forces de frictions, pression, cisaillement…) semblent être la cause principale de la dégénérescence du cartilage articulaire [5]. Cliniquement, douleurs et limitations des mouvements sont les signes essentiels de l'arthrose. La douleur est typiquement une douleur d'effort, de mise en charge [6]. Sur le bilan radiologique, les signes cardinaux classiques de l'arthrose sont une sclérose sous-chondrale plus ou moins associée à des géodes, une ostéophytose implantée à la jonction os-cartilage et un pincement articulaire longtemps localisé. Ce dernier signe est d'interprétation délicate à l'articulation temporo-mandibulaire en raison de l'existence du disque [6]. L'ankylose de l'ATM n'apparait que dans les formes invalidantes, avancées de l'arthrose, dans lesquelles la destruction articulaire est importante radiologiquement et dans lesquelles le traitement médical se révèle insuffisant [2,6]. Jusqu'à l'heure actuelle les publications concernant le lien entre l'arthrose et l'ankylose de l'ATM restent assez rares. Nous pensons qu'un diagnostic précoce et une prise en charge adéquate de l'arthrose de l'ATM pourraient prévenir le développement de l'ankylose de l'ATM dont le traitement est invasif. La prise en charge de l'arthrose de l'ATM peut être divisé en méthodes non invasives (médicaments: anti-inflammatories, Myorelaxants, physiothérapie), minimalement invasives (injections intra-articulaire: acide hyaluronique corticosteroides; arthrocentèse; chirurgie arthroscopique), et modalités invasives ou chirurgicales (arthroplastie, ostéotomie). En phase terminale, les modalités dites de “sauvetage ” doivent être envisagées (reconstruction articulaire totale [7]). Dans le stade de l'ankylose de l'ATM, Les deux gestes incontournables de la prise en charge chirurgicale, sont la résection du bloc d'ankylose, et les coronoidectomies [1]. Malgré ce traitement mutilant, la récidive reste la complication la plus fréquente, avec des pourcentages variant de 0 à 37% [7].

## Conclusion

Le diagnostic d'arthrose de l'ATM doit être présent à l'esprit du chaque praticien de la sphère oro-faciale devant une dysfonction chronique des ATM, afin de le dépister précocement et prévenir ainsi l'évolution vers l'ankylose dont le traitement est lourd.

## Conflits d’intérêts

Les auteurs ne déclarent aucun conflit d'intérêts.
